# Evaluation and 1-year follow-up of patients presenting at a Lyme borreliosis expertise centre: a prospective cohort study with validated questionnaires

**DOI:** 10.1007/s10096-024-04770-6

**Published:** 2024-03-16

**Authors:** F. R. van de Schoor, M. E. Baarsma, S. A. Gauw, J. Ursinus, H. D. Vrijmoeth, H. J. M. ter Hofstede, A. D. Tulen, M. G. Harms, A. Wong, C. C. van den Wijngaard, L. A. B. Joosten, J. W. Hovius, B. J. Kullberg

**Affiliations:** 1 Radboudumc, Department of Internal Medicine, Radboud Center for Infectious Diseases (RCI), Radboud Institute of Health Sciences (RIHS), Radboud University Medical Center, Nijmegen, The Netherlands; 2 Center for Experimental and Molecular Medicine, Amsterdam Institute for Infection and Immunology, Amsterdam UMC, University of Amsterdam, Meibergdreef 9, 1105 AZ Amsterdam, the Netherlands; 3 Center for Infectious Disease Control, National Institute for Public Health and the Environment (RIVM), Bilthoven, the Netherlands; 4 Department of Statistics, Informatics and Modeling, National Institute for Public Health and the Environment (RIVM), Bilthoven, the Netherlands

**Keywords:** Lyme disease, Borreliosis, Erythema migrans, *Borrelia*, PTLBS, PTLDS

## Abstract

**Objectives:**

To describe the course of symptoms reported by patients with symptoms attributed to Lyme borreliosis (LB) without being subsequently diagnosed with LB.

**Methods:**

We performed a prospective cohort study with patients presenting at the outpatient clinic of two clinical LB centres. The primary outcome was the prevalence of persistent symptoms, which were defined as clinically relevant fatigue (CIS, subscale fatigue), pain (SF-36, subscale bodily pain), and cognitive impairment (CFQ) for ≥ 6 months and onset < 6 months over the first year of follow-up. Outcomes were compared with a longitudinal cohort of confirmed LB patients and a general population cohort. Prevalences were standardised to the distribution of pre-defined confounders in the confirmed LB cohort.

**Results:**

Participants (*n* = 123) reported mostly fatigue, arthralgia, myalgia, and paraesthesia as symptoms. The primary outcome could be determined for 74.8% (92/123) of participants. The standardised prevalence of persistent symptoms in our participants was 58.6%, which was higher than in patients with confirmed LB at baseline (27.2%, *p* < 0.0001) and the population cohort (21.2%, *p* < 0.0001). Participants reported overall improvement of fatigue (*p* < 0.0001) and pain (*p* < 0.0001) but not for cognitive impairment (*p* = 0.062) during the follow-up, though symptom severity at the end of follow-up remained greater compared to confirmed LB patients (various comparisons *p* < 0.05).

**Conclusion:**

Patients with symptoms attributed to LB who present at clinical LB centres without physician-confirmed LB more often report persistent symptoms and report more severe symptoms compared to confirmed LB patients and a population cohort.

**Supplementary Information:**

The online version contains supplementary material available at 10.1007/s10096-024-04770-6.

## Introduction

Even though Lyme borreliosis (LB, also known as Lyme disease/LD) generally has a good prognosis, persistent symptoms are reported by a substantial subset of patients [1]. Persistent symptoms after treatment for LB may be persistent objective signs, such as persistent atrophy of the skin associated with acrodermatitis chronica atrophicans (ACA) or neurological damage after Lyme neuroborreliosis, or may be associated with an ongoing aberrant immune response, as has been described for antibiotic-refractory Lyme arthritis [2]. In rare cases, signs may be indicative of a persistent infection with *Borrelia* spirochetes [3]. There is also a group of patients who have more general persistent symptoms (e.g., myalgia, arthralgia, and fatigue) following antibiotic treatment and resolution of the original signs of the LB manifestation. These symptoms are generally classified as Post-Treatment Lyme Borreliosis Syndrome (PTLBS, also known as PTLDS), a suspected post-infectious syndrome of which the exact aetiology has not been ascertained [4]. Yet, others suffer from chronic symptoms that lack a clear explanation by current medical standards, but that are attributed to an (unnoticed) LB episode or a tick bite, despite absent LB-specific symptoms or laboratory confirmation. Regardless of the exact aetiology, these symptoms can seriously affect quality of life [5]. Indeed, PTLBS and chronic symptoms attributed to LB are likely to have the greatest impact on disability-adjusted life years of all LB-related disease categories [5].

A large prospective study in the Netherlands recently showed that the prevalence of various persistent symptoms was 6% higher in confirmed LB patients than in the general population [1]. While there is a growing body of knowledge about persistent symptoms after a confirmed LB diagnosis [1, 6], little is known about the development of symptoms and quality of life in patients that present at clinical LB centres with long-lasting symptoms that are attributed to an unconfirmed LB episode. Studies from Europe [7–14] and the United States [15–17] suggest that LB diagnoses can often not be confirmed in these cases, despite extensive diagnostic testing. The array of persistent symptoms in these patients is sometimes explained by LB, sometimes by alternative diagnoses, and in many cases not explained at all [7–17]. As far as we are aware, no studies have systematically assessed the evolution of these symptoms over time through validated questionnaires in a cohort of patients presenting at a clinical LB centre without being subsequently diagnosed with LB. It is therefore currently unclear how such patients fare in the months after their consultation.

For these reasons, we describe a prospectively included cohort of patients from two clinical LB centres in the Netherlands, performing various clinical measurements, and a follow-up of 1 year using validated questionnaires.

## Methods

### Study design

We conducted a prospective observational cohort study, as previously described [18]. All patients who had a first consultation for clinical care at the outpatient clinic of the LB expertise centres of the Radboud University Medical Centre (Nijmegen, the Netherlands) or Amsterdam University Medical Centre (Amsterdam, the Netherlands) between March 2019 and March 2020 were invited to participate in this study in selected periods of time. Exclusion criteria were the inability to give informed consent or insufficient proficiency in the Dutch language.

### Clinical and laboratory measurements

Clinical measurements were performed at every patient visit and encompassed patients’ signs and symptoms, characteristics of any current or past LB manifestation, results of previous diagnostic tests for LB, prior antibiotic treatment, concomitant medication, and relevant past medical history. To rule out other diagnoses, patients underwent standardised workup by an internal medicine specialist, and by additional medical specialties if needed. A thorough physical examination, including comprehensive neurological assessments, was conducted.

All patients underwent serological testing for *Borrelia*. If necessary, we performed an analysis of cerebrospinal fluid, analysis for Lyme neuroborreliosis (LNB), skin biopsies for cutaneous manifestations, or arthrocentesis to assess Lyme arthritis. Exploratory laboratory tests comprised at least a complete blood count; assessment of thyroid, renal, and hepatic function; and evaluation of inflammatory markers. Diagnostic imaging was employed as deemed necessary. Additional tests, such as the exploration of other tick-borne diseases or autoimmune disorders, were conducted if clinically relevant.

For investigational purposes, a baseline sample was obtained for serological and cellular tests from all patients (more details are provided in the supplementary materials).

### Questionnaires

Study participants were asked to fill out an online questionnaire at baseline, and were invited to fill out an additional questionnaire every 3 months thereafter up to 1 year (i.e., at 3, 6, 9, and 12 months). Online questionnaires were sent via the Tekenradar.nl platform [19]. Clinical outcomes throughout this year were determined through the Checklist Individual Strength (CIS, subscale fatigue) [20], the RAND SF-36 Health Status Inventory (SF-36, subscale bodily pain) [21], and the Cognitive Failure Questionnaire (CFQ) [22]. We also systematically screened for concomitant diagnoses using a list of diagnoses adapted from the TiC-P questionnaire [23]. Participants were reminded by phone or email when they did not fill out a questionnaire in time. Questionnaire data from this study’s participants were compared to two cohorts: patients with confirmed LB from the LymeProspect study (*n* = 1084) and persons from the general population (*n* = 1942) [1]. More details are provided in the supplementary materials.

### Classification of patients

Upon completion of the study, patients were classified by FRvdS and MEB (MDs specializing in LB) independently using all available information from their outpatient visits. Disagreements in classification were referred to an expert panel (FRvdS/MEB/BJK/JWH), which then came to a consensus classification. If another diagnosis was identified, a patient was treated appropriately or was referred to appropriate medical care. We classified patients into one of four groups, namely, as having definite or probable LB, as having PTLBS or residual damage after *Borrelia* infection, as having another apparent diagnosis, or not having a known diagnosis to explain the patient’s symptoms. Patients in the first two groups were classified using established guidelines of the European Society of Clinical Microbiology and Infectious Diseases [24].

### Statistical analyses

Questionnaire data were analysed in a similar fashion to those from the LymeProspect study [1, 19] and as described in the supplementary materials. Briefly, we compared the prevalence of persistent symptoms (i.e., for the symptoms of fatigue, cognitive impairment, and pain, a clinically relevant score on ≥ 1 symptom for ≥ 6 months of the year of follow-up) to the confirmed LB and population cohorts from LymeProspect [1]. Missing questionnaire data were substituted; the primary outcome was only determined when questionnaire data from ≥ 2 timepoints were available. Both overall and point prevalences were standardised using the pre-defined confounders’ sex, age, educational level, and self-reported comorbidity. The development of symptom severity over time was investigated using a linear mixed-effects model. Possible predictors for persistent symptoms were assessed with univariate logistic regression analysis. Due to the limited number of events, we then assessed a multivariable model with a backward selection method using at most five predictors, selecting those with the lowest *p*-values.

For other analyses, we used Fisher’s Exact Test or descriptive statistics. Non-questionnaire data were not imputed. Analyses were performed with R (PBC, version 4.3.2, Boston, MA, USA) and SPSS (IBM Corp, Version 28.0, Armonk, NY, USA). Figures were created in GraphPad Prism (GraphPad Software, Version 9.0, Boston, MA, USA).

### Ethics statement

This study was approved by the Medical Ethics Committee of Amsterdam UMC (NL63961.018.18) and conducted according to the principles of the Declaration of Helsinki and applicable Dutch law. Written informed consent was obtained from all study participants before inclusion.

### Patient involvement

Patient representatives were involved in the study design (e.g., the selection of cellular tests) and have commented on the final version of this manuscript. Patient representatives were not involved as study participants.

## Results

### Inclusion

We included 128 patients presenting at the LB outpatient clinic (Figure S1). As patients with definite/probable LB were outside the scope of this study, participants classified as such (*n* = 5) were excluded, leaving an analytic population of 123 participants. Despite frequent reminders, 31 participants filled out insufficient follow-up questionnaires to determine the prevalence of clinically significant symptoms for ≥ 6 months. Thus, the primary outcome could be determined for 92 participants. Questionnaire non-response was not correlated to LB classification (all comparisons *p* > 0.05).

### Baseline characteristics and classification

General characteristics and classification of all participants are displayed in Table 1 and S1. Patients were mostly referred by general practitioners (96/123, 78.0%) and, to a lesser extent, by medical specialists (25/123, 20.3%) or others (2/123, 1.6%). The majority of patients had symptoms for 2 years or more; fatigue was the most common symptom. Diagnoses which are generally considered contested illnesses [25] such as fibromyalgia (2/123, 1.6%), irritable bowel syndrome (4/123, 3.3%), and chronic fatigue syndrome (0/123, 0%) were infrequently reported in past medical histories.

**Table 1:** Baseline characteristics (*n* = 123)

Age (years), median (IQR; range)	47 (34–56; 18–78)
Male sex, *n* (%)	61 (49.6)
Education, *n* (%)	
Vocational/lower	57 (46.3)
Theoretical/higher	51 (41.5)
Unknown	15 (12.2)
Concomitant diagnoses, *n* (%)	
0	26 (21.1)
1	26 (21.1)
≥ 2	56 (45.5)
Unknown	15 (12.2)
Most commonly reported symptoms, *n* (%)	
Fatigue	91 (74.0)
Arthralgia	80 (65.0)
Myalgia	72 (58.5)
Paraesthesia or sensory abnormalities	50 (40.7)
Neurocognitive symptoms	45 (36.6)
Headache	25 (20.3)
Cardiac complaints (e.g., palpitations)	24 (19.5)
Dizziness	20 (16.3)
Psychological complaints	20 (16.3)
Pain (other than neuralgia, myalgia, or arthralgia)	17 (13.8)
Duration of symptoms, *n* (%)	
< 1 month	2 (1.6)
1–3 months	9 (7.3)
3–6 months	8 (6.5)
6–12 months	15 (12.2)
1–2 years	21 (17.1)
2–10 years	51 (41.5)
> 10 years	16 (13.0)
Unknown	1 (0.8)
Tick bite incidence in area of residence^a^, *n* (%)	
Low	52 (42.3)
Medium	41 (33.3)
High	30 (24.4)
EM incidence in area of residence^a^, *n* (%)	
Low	44 (35.8)
Medium	43 (35.0)
High	36 (29.3)
Previously reported tick bite, *n* (%)	60 (48.8)
Previously reported LB episode, *n* (%)	
Confirmed or probable manifestation(s)	39 (31.7)^b^
Possible or unlikely episode(s)	27 (22.0)
None reported	57 (46.3)
Previous antibiotic treatment, *n* (%)	77 (62.6)
Duration of previous treatment in days, median (IQR; range)	28 (10.5–30.0; 2–780)^c^
Classifications at LB expertise centre^d^, *n* (%)	
PTLBS or residual damage after LB	26 (21.1)
Other diagnosis	26 (21.1)
Unknown diagnosis	71 (57.7)

*EM* erythema migrans, *LB* Lyme borreliosis, *PTLBS* post-treatment Lyme borreliosis syndrome

^a^Low/medium/high represent tertiles of tick bite and EM incidence, respectively

^b^ Manifestations: 35 EM, 1 multiple EM, 3 acrodermatitis chronica atrophicans, 2 Lyme neuroborreliosis, and 2 Lyme arthritis. Numbers do not add up to 39 because some patients had more than one manifestation simultaneously, or reported consecutive episodes of LB

^c^Based on *n* = 69. Eight participants could not accurately report duration of previous antibiotic treatment

^d^Independent assessors agreed on the classification in 109/123 (88.6%) of cases; disagreements were resolved in the expert panel

The majority of patients had received a positive serological test result for LB at some point in the past (77/123, 62.6%). There were 22 patients (17.9%) who had previously sought out a non-recommended diagnostic test for LB, such as cellular tests (*n* = 8), live-blood analysis (*n* = 2), CD57 + measurement (*n* = 2), bioresonance (*n* = 4), and VEGA tests (*n* = 3). Table S2 shows the reactivity of serological and cellular tests for this cohort of patients at baseline. Cellular tests had more missing data due to invalid test results or test samples that were not processed because of shipment delays [26].

Twenty-six patients (21.1%) were classified as having PTLBS or residual damage. Another 26 patients (21.1%) were classified as having another diagnosis (e.g., multiple sclerosis, Guillain-Barré Syndrome, type 2 diabetes, HLA-B27-associated spondyloarthritis, or erythema annulare). No diagnosis was made for the remaining 71 patients (57.7%) (Table 1).

### Follow-up during first year

**Fig. 1:**
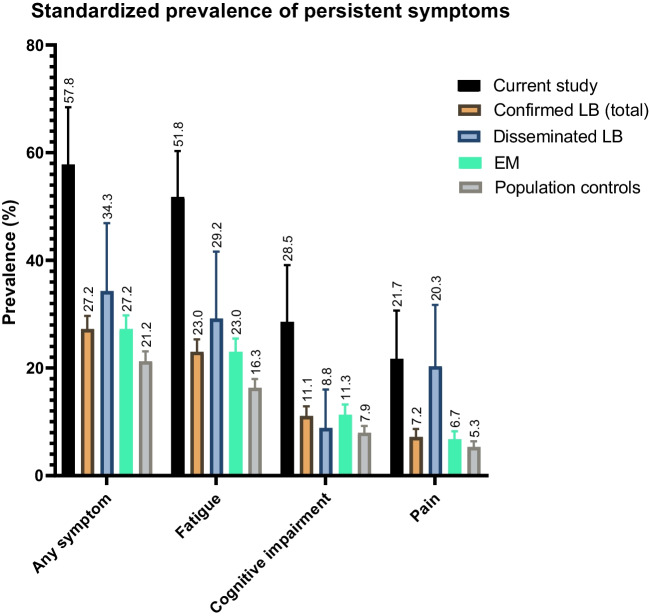
Standardized prevalence of persistent symptoms in participants from the current study (*n* = 92), patients with confirmed LB from the LymeProspect study (*n* = 1084, of whom 1026 with EM and 58 with disseminated LB), and the population cohort (*n* = 1942). Persistent symptoms are defined as clinically relevant fatigue (CIS—subscale fatigue), cognitive impairment (CFQ) and/or pain (SF-36—subscale bodily pain) for ≥ 6 months in the first year of follow-up. Annotated numbers indicate prevalence and error bars represent upper limits of the 95% confidence interval

**Fig. 2:**
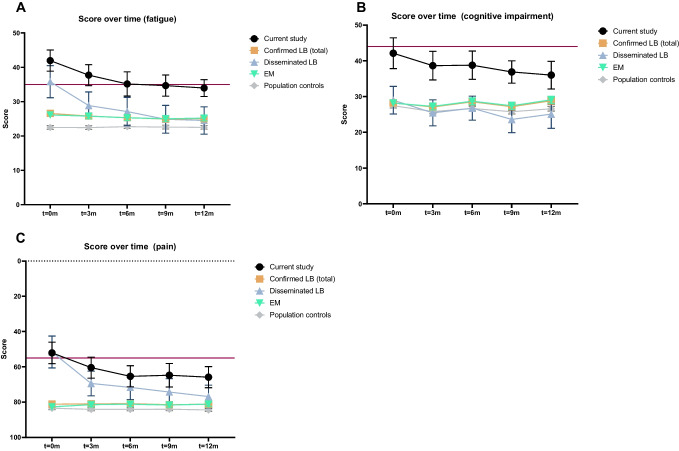
Symptom severity over time, expressed as the standardized mean score in participants from the current study (*n* = 92), in patients with confirmed LB from the LymeProspect study (*n* = 1084, of whom 1026 with EM and 58 with disseminated LB), and in population controls from the LymeProspect study (*n* = 1942). Error bars represent the 95% confidence interval of the standardized mean. The red line indicates the cutoff for a clinically significant score. **A** fatigue (CIS—subscale fatigue), **B** cognitive impairment (CFQ), and **C** pain (SF36—subscale bodily pain)

Of the 92 participants for whom the primary outcome could be assessed, 66 (71.7%) had persistent symptoms as defined above. This crude prevalence was standardised as described and was then compared to patients with a previous episode of confirmed LB and the population cohort [1]. The standardised prevalence of persistent symptoms in the present study was 58.6% (95%CI, 48.3–68.9), which compared unfavourably to patients with previous confirmed LB (27.2%, *p* < 0.0001) and to the population cohort (21.2%, *p* < 0.0001) (Fig. 1). Fatigue was the most prevalent symptom in all groups, followed by cognitive impairment and pain. As shown in Fig. 2A–C and Table S3, the baseline severities of all three symptoms were significantly greater in the study group than in the EM and population cohorts (all comparisons at t = 0 m, *p* < 0.0001). Compared to patients with disseminated LB, the study participants had a significantly higher symptom severity at baseline for fatigue (*p* = 0.0036) and cognitive impairment (*p* < 0.0001) but not for pain (*p* = 0.92).

Study subjects showed improvement over time when using the population controls as a reference (Fig. 2A–C and Table S3), as had been previously described for the confirmed LB cohorts [1, 27]. The improvement was significant for fatigue (*p* < 0.0001) but not for cognitive impairment (*p* = 0.062). For pain, the scores first improved significantly, but this improvement stagnated towards the end of follow-up (Fig. 2C and Table S3). The observed improvements over time in the study group were comparable with the disseminated LB patients from the LB cohort for fatigue and cognitive impairment, but not for pain, because of the stagnation in the improvement of pain in the study group towards the end of follow-up (Fig. 2A–C and Table S3). In spite of these improvements, symptom severity at 12 months was greater in the study subjects than in confirmed LB (Table S3).

**Fig. 3:**
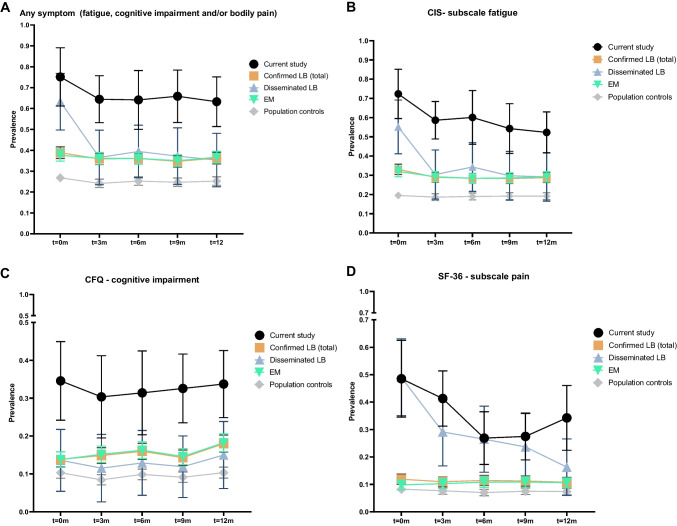
Standardised point prevalence of various clinically relevant symptoms in participants from the current study (*n* = 92), patients with confirmed LB from the LymeProspect study (*n* = 1084, of whom 1026 with EM and 58 with disseminated LB), and population cohort (*n* = 1942). Percentages are expressed as proportions of 1. Error bars represent the 95% confidence interval. **A** any symptom (fatigue, cognitive impairment, and/or bodily pain), **B** fatigue, **C** cognitive impairment, and **D** bodily pain

The improvements in symptom severity translated into a comparable decrease in the standardised prevalence of clinically relevant symptoms over time (Fig. 3A–D). Those patients who were classified as having persistent symptoms had a consistently high symptom severity throughout the follow-up period (Figure S2A–C). There were no apparent differences between the classification-based subgroups in terms of the prevalence of clinically relevant symptoms (Figure S3A–D) or symptom severity (Figure S4A–C).

### Factors related to persistent symptoms

Possible predictors for persistent symptoms were first assessed using univariate logistic regression analyses (Table S4). In the subsequent multivariable model, only lower/vocational education (OR = 3.415; 95%CI, 1.180–9.877; *p* = 0.023) and duration of symptoms > 2 years (OR = 3.155; 95%CI, 1.131–8.800; p = 0.028) remained significant predictors for persistent symptoms.

## Discussion

Our results suggest that symptoms attributed to LB are varied and often clinically relevant. The most common symptoms (fatigue, arthralgia, myalgia, paraesthesia, and neurocognitive symptoms) are frequently reported by patients as being LB-associated. This has previously been described in reports from clinical LB centres [1, 7–17] and in qualitative studies on chronic symptoms attributed to LB [28, 29]. These symptoms are also prevalent in the general population [1, 30, 31]. It is therefore difficult to confirm a relationship between symptoms and a current or past LB episode, in the absence of any specific symptoms or laboratory indication of an active *Borrelia* infection. However, irrespective of their precise aetiology, these symptoms have a substantial impact on the quality of life. The crude observed prevalence of clinically relevant symptoms for ≥ 6 months in the first year of follow-up in our cohort was 71.7%. In order to compare the rate of persistent symptoms with that of confirmed LB patients and the population cohort, we standardised for pre-defined possible confounders such as age, sex, comorbidity, and education level with patients with previously confirmed LB as a reference. This analysis showed that our participants compare unfavourably to patients with a confirmed LB episode and the general population.

Analyses of point prevalences and mean scores at each time point suggest that there is some improvement in the first year after inclusion for both the patients from the current study and those with confirmed LB. Similar improvement was also observed in a comparable cohort of patients [32]. Any improvement in symptoms is most often observed in the first 3 to 6 months after the first consultation and stagnates thereafter. This might partly be explained by regression towards the mean rather than by any interventions [33], i.e., it could be due to the natural course of these symptoms, as patients may be referred to a clinical LB centre when their symptoms are at a peak. In spite of this mean improvement, symptom severity LB at the end of the 12-month follow-up period remained significantly higher in the patients who participated in the current study than in those with confirmed LB.

No obvious predictors for a favourable outcome related to patient management (e.g., antibiotic treatment) were identified in our cohort, with the caveat that our study was not designed to assess the efficacy of any interventions and we had only a limited number of events with which to assess potential predictors. Our exploratory results do suggest that patients with a longer duration of symptoms before inclusion were less likely to recover in the first year of follow-up. This observation is in line with previous research on Medically Unexplained Symptoms (MUS, also known as Persistent Physical Symptoms or PPS) [34, 35] and previous studies on patients from LB expertise centres [32, 36].

Our results also show that there is currently no place for cellular tests in the diagnostic repertoire of LB expertise centres. While frequently sought out by patients with chronic symptoms that are attributed to LB, we found that such tests are frequently positive in patients who do not have any LB-related classification and are reactive in these patients at a level similar to healthy controls [37].

In line with previous studies [7–17, 32, 36], we also found that only a handful of patients at our LB expertise centres had a confirmed LB diagnosis to explain their symptoms. About 20% had symptoms that met the definitions of PTLBS or residual symptoms after an LB manifestation. Another 20% had another diagnosis that could explain the symptoms. These patients were referred for appropriate medical care for their diagnosis. This leaves almost 60% of patients for whom no diagnosis was apparent. For some of these patients, their symptoms may be explained by an undiagnosed other condition; for others, they may be entirely functional in nature, and for yet others, they could hypothetically be related to a (past) *Borrelia* infection through some unidentified pathophysiological mechanism. Interestingly, our patients’ heterogeneous symptoms and disease burden frequently permit a differential diagnosis of fibromyalgia, chronic fatigue syndrome, or a general post-infectious syndrome [38–40], sometimes all at the same time. We did not systematically assess whether patients met the criteria for these diagnoses because this was outside the scope of the current study. We also feel compelled to point out that these are all *syndrome diagnoses*, based on clinical presentation but without a known pathophysiological mechanism. These labels frequently overlap; asthenia, for example, can be a symptom of all of the aforementioned diagnoses. A recent paper has suggested the introduction of the term ‘Functional Somatic Disorders’ as an umbrella term for similar symptoms without a known cause [41]. The authors highlight its potential for research and treatment based on shared aetiological mechanisms between syndromes that are currently studied separately and its potential to resolve the historic ‘split’ between a purely organic and purely mental cause of illness [41].

What is abundantly clear is that patients similar to the ones in the current study have the highest disease burden of all patients who are assessed for a potential LB diagnosis, as has been consistently shown in the current and previous research [1, 5, 32, 36]. What many of these patients share is that they explain their illness either entirely or in part through the lens of LB, irrespective of whether current medical science supports that attribution. Healthcare providers should connect with this explanatory model and create common ground with their patients [28]. Thus, they are better suited to provide effective care, through open and frank conversations about the benefits and pitfalls of further LB-related diagnostics and treatments, all with the goal of preventing iatrogenic harm and improving their patients’ health. We encourage continued research on specific treatments for this group, either based on factors specific for a (past) *Borrelia* infection or based on shared aetiological mechanisms for functional disorders. Until then, we will endeavour to provide appropriate supportive care.

Our study may have several limitations. Due to the COVID-19 pandemic, follow-up visits and samples were not available for a number of patients, even though 75.8% of participants did fill out sufficient questionnaires to assess the primary outcome. Our study also does not answer the question of how our participants fare after the first year. We would argue that additional follow-up studies—including those assessing the effect of interventions—on patients with long-lasting LB-associated symptoms are needed. It is difficult to assess with certainty whether we have a representative spectrum of patients, as patients with the most severe symptoms are more likely to enrol in these kinds of studies, which may lead to an underrepresentation of patients who fare better. On a final note, we point out that we performed a thorough clinical work-up to exclude the most likely differential diagnosis, as we described above. However, we cannot guarantee with absolute certainty that all patients without a known diagnosis did not have an organic cause of their illness.

In summary, our results show that the large majority of patients at LB referral clinics suffer from clinically significant symptoms for ≥ 6 months after their consultation, which are frequently not explained by any recognized LB-related disease entity. Our study highlights the suffering of these patients and the need for improved strategies for diagnosis and treatment of their symptoms.

### Supplementary Information

Below is the link to the electronic supplementary material.

Supplementary file1 (DOCX 157 KB)

## Data Availability

The datasets generated and/or analysed during the current study are not publicly available but are available from the corresponding author upon reasonable request.
