# Supplementary material for: Evaluation and 1-year follow-up of patients presenting at a Lyme borreliosis expertise centre: a prospective cohort study with validated questionnaires
Source: Eur J Clin Microbiol Infect Dis. 2024 Mar 16;43(5):937–46. doi: 10.1007/s10096-024-04770-6 (PMC11108889; doi:10.1007/s10096-024-04770-6)
Supplement: Supplementary file 1 — Supplementary file1 (DOCX 157 KB) [file 10096_2024_4770_MOESM1_ESM.docx]

**SUPPLEMENTARY FILE WITH**

**Evaluation and one-year follow-up of patients presenting at a Lyme borreliosis expertise centre: a prospective cohort study with validated questionnaires**

F.R. van de Schoor^1*^, M.E. Baarsma^2*^, S.A. Gauw ^2^, J. Ursinus^2^, H.D. Vrijmoeth^1^, H.J.M. ter Hofstede^1^, Anna D. Tulen^3^, M.G. Harms^3^, A. Wong^4^, C.C. van den Wijngaard^3$^, L.A.B. Joosten^1$^, J.W. Hovius^2$^, B.J. Kullberg^1$^

* Shared first authorship | $ Shared senior authorship

**Affiliations**

1. Radboudumc, Department of Internal Medicine, Radboudumc Center for Infectious Diseases (RCI) and Radboud Institute of Health Sciences (RIHS), Nijmegen, The Netherlands
2. Amsterdam UMC, University of Amsterdam, Center for Experimental and Molecular Medicine, Amsterdam Institute for Infection and Immunology, Amsterdam, The Netherlands
3. National Institute for Public Health and the Environment (RIVM), Center for Infectious Disease Control, Bilthoven, The Netherlands
4. National Institute for Public Health and the Environment (RIVM), Department for Statistics, Informatics and Modeling, Bilthoven, The Netherlands

**SUPPLEMENTARY MATERIALS 1 - METHODS**

**Execution and interpretation of laboratory tests**

Standard two-tier serological testing was performed using the C6-ELISA (Oxford Immunotec, Oxford, United Kingdom) in the first tier. Sera with an equivocal or positive result in the first tier were tested using recomLine IgM and/or IgG immunoblots (Mikrogen GmbH, Neuried, Germany). Results for the recomLine immunoblots were taken from two sources. If an immunoblot result for a given participant was available through routine clinical diagnostics, then that blot was not performed again but the result from routine diagnostics was used for study purposes. Other sera were tested for study purposes specifically.

In addition, we used three cellular tests for LD: the Spirofind Revised (Oxford Immunotec, Oxford, UK), the commercially available Lyme iSpot (Autoimmun Diagnostika, Strassberg, Germany), and LTT-MELISA (InVitaLab, Neuss, Germany). The Spirofind was performed at Amsterdam UMC and Radboudumc and was interpreted as prescribed by the manufacturer. Samples for the iSpot and [LTT](https://www.sciencedirect.com/topics/medicine-and-dentistry/lymphocyte-transformation) were transferred to the facilities of their respective manufacturers in Germany by overnight courier to be assessed there.

The results of individual tests are reported in a primary and an alternate interpretation. For the iSpot, LTT and C6-ELISA, equivocal results were classified as positive in the primary interpretation and classified as negative in the alternate interpretation. For the Spirofind, which does not have any equivocal results by design, the primary interpretation included only samples that were processed strictly according to the manufacturer’s protocol, while the alternate interpretation included all samples that had an interpretable result, even those with a protocol deviation (e.g., exceeding permitted time from blood draw to start of incubation). An immunoblot was deemed positive when either the IgM, the IgG or both components were positive; all other combinations were classified as negative.

The study originally included a fourth cellular test (the QuantiFERON-Lyme, QIAGEN, Germantown, Maryland, USA). As preliminary results for this assay showed a near-complete lack of reactivity in patients with confirmed LB, we did not perform this assay in the cohort described in the current manuscript. The aforementioned preliminary results have been published elsewhere (1).

**Questionnaire data: general description of statistical analyses**

Questionnaire data were analysed in a similar fashion as questionnaire data from the LymeProspect study (2, 3), of which an updated description is given below.

As mentioned in this manuscript’s main text, data were used from this study’s participants, as well as from two cohorts from the LymeProspect study: patients with confirmed LB (EM and disseminated LB), and controls from the general population. All participants from the aforementioned groups who had completed the primary outcome questionnaires (CIS - subscale fatigue; CFQ; and SF-36 - subscale pain) at ≥1 time point were included in the analyses. The prevalence of persistent symptoms in all cohorts was assessed with a primary analysis scenario for substitution of missing data, if primary outcome questionnaires were completed at ≥2 time points. Missing scores were substituted by linear interpolation of available preceding and following continuous scores of the particular questionnaire. Missing first or final observations were substituted by carrying backward the first or forward the last available questionnaire score. Primary outcomes were standardized to the distribution of the pre-defined confounders sex, age, educational level and self-reported comorbidity in the confirmed LB cohort from LymeProspect (see below). Because of low numbers of patients with disseminated LB, only sex and comorbidity were used as confounders for analysis in those groups.

Severity of symptoms was assessed by evaluating the mean questionnaire score for each symptom at baseline and during follow-up. Symptom severity over time of this study’s participants was compared with the severity in the confirmed LB patients and controls using linear mixed effects models, where the score was considered a function dependent on cohort, time and confounders (see below). In addition, differences in mean severity scores at the end of follow-up were assessed between cohorts.

For the analyses of severity and prevalence of impaired questionnaire scores at individual time points, missing questionnaire scores were not substituted and standardization was performed for each specific time point. Differences in the prevalence of persistent symptoms and in severity scores at the 12 months’ time point between cohorts were assessed using permutation tests.

**Questionnaire data: predefined confounders and permutation tests**

To reduce confounding effects as much as possible, indirect standardization was performed with respect to this study’s participants and the cohorts from the LymeProspect study (3). To this end, strata were formed using predefined confounders (age, sex, educational level and comorbidity). The continuous confounders were coded as categorical variables, by using breaks that were chosen after exploring the crude prevalence of persistent symptoms in the LB patients and control cohorts from LymeProspect, based on the primary scenario for substitution for missing data. This led to the following categorical variables:

- Age: breaks at age 45 and 65 years.
- Sex: male and female.
- Educational level: eight Dutch educational levels were divided into two categories, vocational/low and theoretical/high educational level, to limit the number of strata. University of applied sciences and academic university education were categorized as theoretical/high educational level; others as vocational/low.
- Comorbidity: based on the number of reported comorbidities - as listed by the TiC-P questionnaire (4) - three categories were included (0, 1 and ≥2 comorbidities).

Only two confounders were used in the analyses of patients with disseminated LB, because of the low numbers of this participant type. These were sex (male/female), and comorbidity in two categories (0 and ≥1 comorbidities).

Within each cohort and stratum, a sample mean and variance of the mean was determined. These were pooled to an overall mean and variance of the mean, weighted to the relative size of the stratum (mean) and the square of the relative size (variance of the mean) within the LB patients. The 95% confidence intervals were computed through normal approximations from the pooled variances of the mean. For the comparison of the prevalence of persistent symptoms between this study’s participants, the confirmed LB patients and the population controls, permutation tests based on the sum statistic were used with the same confounders as for the indirect standardization (5, 6). If strata only contained subjects from one cohort, they were omitted from testing, leading to lower sample sizes.

**Questionnaire data: linear mixed effects models for assessment of differences in severity over time**

Linear mixed effects models were used to construct a standardized symptom severity course and to assess differences in this course between cohorts. The outcome of this model is a function dependent on an intercept, cohort, a quadratic polynomial for time, an interaction between cohort and that polynomial, and the used confounders. A random effect for each individual was used to account for the correlation between observations within an individual. If any p-values for the cohort fixed effects or interactions were significant, the overall course of symptom severity in the assessed cohort was considered to be different from the reference cohort. In the primary analysis, this study’s participants were compared to patients with confirmed LB from LymeProspect, and that study’s population controls.

**SUPPLEMENTARY MATERIALS 2 - RESULTS**

**FIGURE S1 –** Flowchart of participants of the LymeProspect study (7) and the current study. Abbrevations: PTLBS: Post-treatment Lyme borreliosis Syndrome; EM: erythema migrans.

**66 patients with persistent symptoms**

- 16 PTLBS / residual damage
- 37 Unknown diagnosis
- 13 Other diagnosis

**26 patients without persistent symptoms**

- 6 PTLBS / residual damage
- 13 Unknown diagnosis
- 7 Other diagnosis

**Patients visiting Lyme borreliosis expertise centres**

Current study

31 did not fulfill criteria for the analysis of primary outcome

5 patients probable/proven LB excluded from analysis

**92 patients included in the analysis**

- 22 PTLBS / residual damage
- 50 Unknown diagnosis
- 20 Other diagnosis

**123 patients without proven/probable LB**

- 26 PTLBS / residual damage
- 71 Unknown diagnoses
- 26 Other diagnosis

**128 inclusions**

**Population cohort**

**Lyme borreliosis patients**

**1942 participants included in the analysis**

2058 did not fulfill criteria for the analysis of primary outcome

86 patients excluded from analysis

**4000 individuals**

**4086 inclusions**

**1084 patients included in the analysis**

51 did not fulfill criteria for the analysis of primary outcome

43 patients excluded from analysis

**1135 Lyme borreliosis patients**

- 1076 erythema migrans
- 30 acrodermatitis chronica atrophicans
- 16 Lyme neuroborreliosis
- 12 Lyme arthritis
- 1 borrelial lymphocytoma
- 1 early (sub)acute symptoms without EM

**1178 inclusions**

LymeProspect study

| **TABLE S1: Baseline characteristics of the current study compared to the study of Ursinus and colleagues (7), applicable parameters only** | | | | | |
| --- | --- | --- | --- | --- | --- |
| **Characteristic** | **The current study (n = 123)** | **Population cohort (n = 4000)** | **All LB patients (n = 1135)** |  | |
|  |  |  |  | **EM (n = 1076)** | **Disseminated LB (n = 59)** |
| **Male sex - no. (%)** | 61 (49.6) | 1892 (47.3) | 465 (41.0) | 432 (40.1) | 33 (55.9) |
| **Age (years)** | 47.0 (34-56) | 57 (50–63) | 55 (45–63) | 55 (45–63) | 53 (47–63) |
| **Educational level - no. (%)** Low High | 57 (52.8)  51 (47.2) | 2475 (61.9) 1525 (38.1) | 516 (45.5) 619 (54.5) | 485 (45.1) 591 (54.9) | 31 (52.5) 28 (47.5) |
| **Concomitant diagnoses (past 12 months)** Yes - no. (%) Number of diagnoses | 82 (75.9)**^b^**  2 (1-2) | 2078 (51.9) 1 (0–1) | 613 (54.0) 1 (0–1) | 577 (53.6) 1 (0–1) | 36 (61.0) 1 (0–2) |
| **Lyme borreliosis in history - no. (% of total cohort)** | 39 (31.7) | 253 (6.3) | 101 (8.9) | 94 (8.7) | 7 (11.9) |
| **Noticed tick bite - no. (%)** | 60 (48.8) | N/A | 663 (58.8) | 647 (60.6) | 16 (27.1) |
| **Duration of LB manifestation (days)** | N/A | N/A | 6 (3–18) | 5 (3–14) | 208 (50–665) |
| Differences between participants in the current study and the population control/LB cohorts from the study by Ursinus and colleagues were corrected in the primary analyses by standardization for age, sex, comorbidity and educational level. Continuous variables are reported as median (IQR). For calculating percentages, missing data were excluded. a. Fifteen participants of the current study did not report educational level. b. Fifteen participants did not report whether they had any concomitant diagnoses. *Abbreviations:* LB: Lyme borreliosis; EM: erythema migrans; N/A: not available. | | | | | |

| **TABLE S2: Reactivity in serological and cellular tests** | | | | | | | | |
| --- | --- | --- | --- | --- | --- | --- | --- | --- |
| **Baseline** | **PTLBS or residual damage**  **(n=26)** | | **Other diagnosis**  **(n=26)** | | **Unknown diagnosis**  **(n=71)** | | **Healthy controls**  **(n=228)^a^** | |
|  | No. (%) | 95%CI | No. (%) | 95%CI | No. (%) | 95%CI | No. (%) | 95%CI |
| Spirofind – primary | 7/23 (30.4) | 13.2-52.9 | 4/19 (21.1) | 6.1-45.6 | 13/60 (21.7) | 12.1-34.2 | 31/171 (18.1) | 12.7-24.7 |
| Spirofind – alternate | 9/25 (36.0) | 18.0-57.5 | 4/24 (16.7) | 4.7-37.4 | 14/64 (21.9) | 12.5-34.0 | 44/218 (20.2) | 15.1-26.1 |
| iSpot Lyme – primary | 4/10 (40.0) | 12.2-73.8 | 9/10 (90.0) | 55.5-99.8 | 25/38 (65.8) | 48.7-80.4 | 71/103 (68.9) | 59.1-77.7 |
| iSpot Lyme – alternate | 2/10 (20.0) | 2.5-55.6 | 5/10 (50.0) | 18.7-81.3 | 14/38 (36.8) | 21.8-54.0 | 24/103 (23.3) | 15.5-32.7 |
| LTT-MELISA – primary | 11/20 (55.0) | 31.5-76.9 | 14/22 (63.6) | 40.7-82.8 | 23/60 (38.3) | 26.1-51.8 | 90/190 (47.4) | 40.1-54.7 |
| LTT-MELISA – alternate | 8/20 (40.0) | 19.1-64.0 | 10/22 (45.5) | 24.4-67.8 | 20/60 (33.3) | 21.7-46.7 | 60/190 (31.6) | 25.0-38.7 |
| C6-ELISA – primary | 13/26 (50.0) | 29.9-70.0 | 13/26 (50.0) | 29.9-70.0 | 30/71 (42.3) | 30.6-54.6 | 16/228 (7.0) | 4.1-11.2 |
| C6-ELISA - alternate | 12/26 (46.2) | 26.6-66.6 | 11/26 (42.3) | 23.4-63.1 | 28/71 (39.4) | 28.0-51.8 | 14/228 (6.1) | 3.4-10.1 |
| STTT | 9/26 (34.6) | 17.2-55.7 | 8/26 (30.8) | 14.3-51.8 | 23/71 (32.4) | 21.7-44.6 | 12/228 (5.3) | 2.8-9.0 |
| Abbreviations: LB: Lyme borreliosis; PTLBS: post-treatment Lyme borreliosis syndrome; 95%CI: 95% confidence interval  This table omits tests results that were missing or non-valid for any reason. The primary and alternate interpretations of each test are:  - Spirofind (primary): including only those samples performed strictly according to protocol; Spirofind (alternate): including all tests with an interpretable result  - iSpot Lyme/LTT-MELISA/C6-ELISA (primary): counting equivocal results as positive; iSpot Lyme/LTT-MELISA/C6-ELISA (alternate) counting equivocal results as negative  ^a^ Data taken from related study (8) and shown for comparison. | | | | | | | | |

**FIGURE S2A-C** – Symptom severity over time, expressed as the standardized mean score in participants from the current study with persistent symptoms for ≥6 months after baseline and those without persistent symptoms. Error bars represent the 95% confidence interval of the standardized mean. Red line indicates the cut-off for a clinically significant score. | **A** – fatigue (CIS – subscale fatigue) | **B** – cognitive impairment (CFQ) | **C** – pain (SF36 – subscale bodily pain), y-axis inverted, a lower score indicates more severe symptoms.

| **TABLE S3: Statistical tests based on linear mixed effects model and permutation tests** | | | | | |
| --- | --- | --- | --- | --- | --- |
| **Cohort** | **Reference cohort** | **Model parameter** | **p-value**  **CIS** | **p-value**  **CFQ** | **p-value**  **SF-36** |
| Symptom severity at baseline | | | | | |
| Study group | Population cohort | Study group | <0.0001 | <0.0001 | <0.0001 |
|  | EM patients | Study group | <0.0001 | <0.0001 | <0.0001 |
|  | Disseminated LB | Study group | 0.0036 | <0.0001 | 0.92 |
| Symptom severity over time | | | | | |
| Study group | Population cohort | Time point and  Study group | <0.0001 | 0.062 | <0.0001 |
|  |  | Square time point and Study group | 0.0024 | 0.96 | <0.0001 |
|  | EM patients | Time point and  Study group | 0.0005 | 0.021 | <0.0001 |
|  |  | Square time point and Study group | 0.031 | 0.68 | <0.0001 |
|  | Disseminated LB | Time point and  Study group | 0.22 | 0.77 | 0.046 |
|  |  | Square time point and Study group | 0.53 | 0.41 | 0.47 |
| Symptom severity at t=12m | | | | | |
| Study group | Population cohort | Study group | <0.0001 | <0.0001 | <0.0001 |
|  | EM patients | Study group | <0.0001 | 0.0047 | <0.0001 |
|  | Disseminated LB | Study group | <0.0001 | 0.016 | 0.0010 |
| *Abbreviations:* CIS: Checklist Individual Strength – subscale fatigue; CFQ: Cognitive Failure Questionnaire; SF-36: Short Form Health Survey – subscale bodily pain; EM: erythema migrans; LB: Lyme borreliosis | | | | | |

**FIGURE S3A-D** – Standardised point prevalence of various clinically relevant symptoms in participants from the current study, classified as having PTLBS/residual damage, as having another diagnosis, or as having no known diagnosis. Percentages are expressed as proportions of 1. Error bars represent the 95% confidence interval. | **A** – any symptom (fatigue, cognitive impairment and/or bodily pain) | **B** – fatigue | **C** – ****cognitive impairment | **D** – bodily pain

**FIGURE S4A-C –** Symptom severity over time, expressed as the standardized mean score in participants from the current study, classified as having PTLBS/residual damage, as having another diagnosis, or as having no known diagnosis. Error bars represent the 95% confidence interval of the standardized mean. Red line indicates the cut-off for a clinically significant score. | **A** – fatigue (CIS – subscale fatigue) | **B** – cognitive impairment (CFQ) | **C** – pain (SF36 – subscale bodily pain) , y-axis inverted, a lower score indicates more severe symptoms.

| **TABLE S4: Univariate logistic regression analysis for persistent symptoms** | | | | | |
| --- | --- | --- | --- | --- | --- |
| **Characteristic** | **Reference group** | **OR** | **95%CI** | **p-value** | **N** |
| Male sex | Female sex | 0.593 | 0.238-1.480 | 0.263 | 92 |
| Age | *Continuous* | 0.972 | 0.937-1.008 | 0.121 | 92 |
| Higher/theoretical education | Lower/vocational education | 0.271 | 0.100-0.734 | 0.010 | 92 |
| Comorbidity ≥2 diagnoses | 1. No diagnoses  2. 1 diagnosis | 2.871 | 1.094-7.530 | 0.032 | 92 |
| No. of symptoms | *Continuous* | 1.319 | 1.050-1.657 | 0.017 | 92 |
| Duration of symptoms ≥2y | Symptoms <2y | 3.227 | 1.245-8.361 | 0.016 | 91 |
| Length of prior antibiotic treatment | *Continuous* | 0.998 | 0.983-1.012 | 0.736 | 88 |
| PTLDS and residual damage | 1. Other diagnosis  2. Unknown diagnosis | 1.067 | 0.365-3.116 | 0.906 | 92 |
| *Abbreviations:* OR: odds ratio; 95%CI: 95%CI confidence interval; LB: Lyme borreliosis; PTLBS: post-treatment Lyme borreliosis syndrome | | | | | |

**REFERENCES FOR THIS SUPPLEMENTARY FILE**

1. Baarsma ME, van de Schoor FR, Van den Wijngaard CC, Joosten LAB, Kullberg BJ, Hovius JW. The Initial QuantiFERON-Lyme Prototype is Unsuitable for European Patients. Clin Infect Dis. 2021;73(6):1125-6.

2. Vrijmoeth HD, Ursinus J, Harms MG, Zomer TP, Gauw SA, Tulen AD, et al. Prevalence and determinants of persistent symptoms after treatment for Lyme borreliosis: study protocol for an observational, prospective cohort study (LymeProspect). BMC Infect Dis. 2019;19(1):324.

3. Ursinus J, Vrijmoeth HD, Harms MG, Tulen AD, Knoop H, Gauw SA, et al. Prevalence of persistent symptoms after treatment for lyme borreliosis: A prospective observational cohort study. The Lancet Regional Health – Europe. 2021;6.

4. Bouwmans C, De Jong K, Timman R, Zijlstra-Vlasveld M, Van der Feltz-Cornelis C, Tan Swan S, Hakkaart-van Roijen L. Feasibility, reliability and validity of a questionnaire on healthcare consumption and productivity loss in patients with a psychiatric disorder (TiC-P). BMC Health Serv Res. 2013;13:217.

5. Hothorn T, Hornik K, van de Wiel MAV, Zeileis A. Implementing a Class of Permutation Tests: The coin Package. J Stat Softw. 2008;28(8):1-23.

6. Ferreira JA. Some models and methods for the analysis of observational data. Statistics Surveys. 2015;9:106-208.

7. Ursinus J, Vrijmoeth HD, Harms MG, Tulen AD, Knoop H, Gauw SA, et al. Prevalence of persistent symptoms after treatment for lyme borreliosis: A prospective observational cohort study. Lancet Reg Health Eur. 2021;6:100142.

8. Baarsma ME, van de Schoor FR, Gauw SA, Vrijmoeth HD, Ursinus J, Goudriaan N, et al. Diagnostic parameters of cellular tests for Lyme borreliosis in Europe (VICTORY study): a case-control study. Lancet Infect Dis. 2022;22(9):1388-96.
